# Integrating genome-wide DNA methylation and mRNA expression profiles identified different molecular features between Kashin-Beck disease and primary osteoarthritis

**DOI:** 10.1186/s13075-018-1531-1

**Published:** 2018-03-07

**Authors:** Yan Wen, Ping Li, Jingcan Hao, Chen Duan, Jing Han, Awen He, Yanan Du, Li Liu, Xiao Liang, Feng Zhang, Xiong Guo

**Affiliations:** 1 Key Laboratory of Trace Elements and Endemic Diseases of National Health and Family Planning Commission, School of Public Health, Xi’an Jiaotong University, Health Science Center, No.76 Yan Ta West Road, Xi’an, 710061 People’s Republic of China; 2 The First Affiliated Hospital of Xi’an Jiaotong University, Xi’an, 710061 People’s Republic of China

**Keywords:** Kashin-Beck disease, Osteoarthritis, Methylation, Gene expression

## Abstract

**Background:**

Kashin-Beck disease (KBD) is an endemic osteochondropathy of unknown etiology. Osteoarthritis (OA) is a form of degenerative joint disease sharing similar clinical manifestations and pathological changes to articular cartilage with KBD.

**Methods:**

A genome-wide DNA methylation profile of articular cartilage from five KBD patients and five OA patients was first performed using the Illumina Infinium HumanMethylation450 BeadChip. Together with a previous gene expression profiling dataset comparing KBD cartilage with OA cartilage, an integrative pathway enrichment analysis of the genome-wide DNA methylation and the mRNA expression profiles conducted in articular cartilage was performed by InCroMAP software.

**Results:**

We identified 241 common genes altered in both the DNA methylation profile and the mRNA expression profile of articular cartilage of KBD versus OA, including CHST13 (NM_152889, fold-change = 0.5979, *P*_*methy*_ = 0.0430), TGFBR1 (NM_004612, fold-change = 2.077, *P*_*methy*_ = 0.0430), TGFBR2 (NM_001024847, fold-change = 1.543, *P*_*methy*_ = 0.037), TGFBR3 (NM_001276, fold-change = 0.4515, *P*_*methy*_ = 6.04 × 10^−4^), and ADAM12 (NM_021641, fold-change = 1.9768, *P*_*methy*_ = 0.0178). Integrative pathway enrichment analysis identified 19 significant KEGG pathways, including mTOR signaling (*P* = 0.0301), glycosaminoglycan biosynthesis-chondroitin sulfate/dermatan sulfate (*P* = 0.0391), glycosaminoglycan biosynthesis-keratan sulfate (*P* = 0.0278), and PI3K-Akt signaling (*P* = 0.0243).

**Conclusion:**

This study identified different molecular features between Kashin-Beck disease and primary osteoarthritis and provided novel clues for clarifying the pathogenetic differences between KBD and OA.

**Electronic supplementary material:**

The online version of this article (10.1186/s13075-018-1531-1) contains supplementary material, which is available to authorized users.

## Background

Kashin-Beck disease (KBD) is an endemic and chronic osteochondropathy in China. According to the China Health Statistic Yearbook in 2013, there are still 645,000 people suffering from KBD. The original pathological changes of KBD appear in the deep zone of the growth plate cartilage and articular cartilage [1], which will result in premature closure of the epiphyseal plate and impaired endochondral ossification. Some genetic factors, such as ITPR2, HLA-DRB1, and ABI3BP, are thought to be causes of KBD and lead to a difference between individuals in the incidence of KBD [2–4].

Primary osteoarthritis (OA) is a form of degenerative joint disease. It is highly prevalent worldwide and affects about 10% of men and 18% of women over 60 years of age [5]. The biological and pathological alterations of OA mainly happen in the cartilage, subchondral bone, and synovium. Recently, systemic inflammation has also been found to be associated with OA [6]. Generally, the etiology of OA is complex and multifactorial, with genetic, biological, and biomechanical components involved.

KBD and OA share common characteristics regarding the manifestation and pathological changes in the articular cartilage. For example, necrosis and apoptosis of chondrocytes can both appear in the articular cartilage of KBD and OA. Additionally, the narrowed joint space, movement disorder of joints, painful joints, and osteophytes can be seen both in KBD and OA cartilage. However, KBD is an endemic disease causing by environmental risk factors. The etiology and molecular mechanism of KBD are different from OA [7], and clarifying the differences between KBD and OA would be helpful for pathogenetic and therapeutic studies of both KBD and OA. Previously, we compared the genome-wide expression profiles of KBD and OA [7]. Recent studies have demonstrated the important role of DNA methylation in the development of OA [8]. Therefore, integrative analysis of genome-wide DNA methylation and mRNA expression profiles may provide novel clues for understanding the pathogenesis of both KBD and OA.

DNA methylation, as an epigenetic control mechanism, plays an important role in the regulation of gene expression and, therefore, various biological processes and diseases. It is tissue-specific, dynamic, and sequence context-dependent [9]; thus, it has great significance in deciphering the complex methylation patterns needed for answering biological questions. Recent studies have demonstrated an association between the epigenetic changes and the progression of OA [8, 10–13]. Shi et al. also found that there is a difference in the methylation status of KBD blood compared to normal blood [14].

Facilitated by recent technological advances, it has become easier and quicker to obtain high-throughput data at one time point regarding the genomic, epigenomic, transcriptomic, and proteomic scales. It is thought that there will be an exciting potential for answering more biological questions by integrating these together [15]; to take DNA methylation as an example, to show that the epigenetic feature can regulate transcriptional results, it will be helpful to correlate the epigenomic data and transcriptomic data. Gao et al. matched DNA methylation data to the RNA-seq data of breast cancer and regarded the epigenetic silencing of WNT signaling antagonists and bone morphogenetic proteins as key events underlying breast cancer [16]. Therefore, it is necessary to conduct an integrative analysis between DNA methylation and gene expression data rather than studying them individually.

In this study, a genome-wide methylation study was conducted on the articular cartilage of KBD and comparing this to OA. Furthermore, by utilizing previously published gene expression profiling study data comparing the articular cartilage of KBD with OA, an integrative analysis was conducted in these two datasets using InCroMAP software. Our results illustrated different molecular features and biological networks underlying KBD compared to OA.

## Methods

### Sample collection

Cartilage specimens were collected from the femoral condyles of knee joints of KBD and OA patients undergoing total knee joint arthroplasty. DNA samples were extracted from the cartilage specimens using QIAamp DNA Mini Kit (QIAGEN, Germany). All study subjects were Chinese Han. KBD donors came from the KBD prevalent area of Yongshou county, Xi’an city of the Shaanxi province, while OA donors were from Xi’an city of the Shaanxi province. Written informed consent was obtained from all subjects. KBD patients were diagnosed strictly according to national diagnostic criteria of Kashin-Beck disease in China (WS/T 207-2010). OA patients were diagnosed strictly according to the Western Ontario and McMaster Universities Osteoarthritis Index (WOMAC). Any subjects who had a history of other bone or joint diseases were excluded from this study.

Finally, five KBD patients (three males and two females) and five OA patients (three males and two females) were collected and divided into five pairs matched according to their age and sex for the DNA methylation study. The average ages of KBD patients and OA patients were 57.4 ± 7.12 and 64.6 ± 5.01 years, respectively.

### Genome-wide DNA methylation study

In this study, the Illumina Infinium HumanMethylation450 BeadChip (Illumina, Inc., USA) was used to carry out the genome-wide DNA methylation study. Genomic DNA was extracted from articular cartilage. A total of 500 ng DNA was used for bisulfite conversion according to the standard protocol for the EZ DNA Methylation Kit (Zymo Research, USA). The DNA solution was mixed with NaOH and then melted into a single-stranded molecular sample. Then, the whole-genome amplification was conducted and the prepared sample was incubated at 37 °C overnight. After hybridization to the HumanMethylation450 array, the array was washed and stained followed the protocol of the Infinium HD Assay Methylation. The fluorescence signal was scanned by the IScan SQ scanner (iScan System, Illumina, USA), and the obtained raw image intensity data were processed using GenomeStudio software (Illumina, USA).

For the genome-wide DNA methylation study, the β value was defined as the expression of the average percentage of methylated cytosine at a given CpG site, which varied from 0 (completely unmethylated) to 1 (completely methylated). Differentially methylated CpG sites were identified using the empirical Bayes moderated *t* test, which was described by the Illumina Methylation Analyzer package. For each CpG site, the false discovery rate (FDR)-adjusted *P* value was calculated using the Benjiamini-Hochberg method. The definition of significant CpG was: 1) FDR-adjusted *P* value ≤ 0.05; and 2) β-value difference (Δβ) ≥ 0.2. For quality control, the samples with more than 90% missing values or detection *P* value > 0.05 in more than 90% of cartilage specimens were eliminated.

### Gene expression profiling study

The dataset of gene expression profiling for KBD and OA from our previous study was used here [7]. Briefly, KBD donors came from the KBD prevalent areas of Yongshou county and Linyou county of Xi’an city of the Shaanxi province, while OA donors were collected from Xi’an city of the Shaanxi province. Cartilage specimens were collected from the femoral condyles of knee joints of four KBD patients (three males and one female) and four OA patients (three males and one female) undergoing total knee joint arthroplasty. The average ages of KBD patients and OA patients were 59 ± 1.63 and 62 ± 2.94 years, respectively. Total RNA was isolated from the patient’s articular cartilage using the Agilent Total RNA Isolation Mini kit (Agilent Technologies, Santa Clara, CA, USA). The integrity and concentration of RNA samples were detected by agarose gel electrophoresis and spectrophotometer. The RNA was reverse-transcribed into cDNA and then the cDNA was transcribed into cRNA in the presence of Cy3- or Cy5-CTP. The labeled cRNA of KBD and OA was purified separately and mixed with hybridization buffer. The hybridization solution was prepared using the In Situ Hybridization Plus Kit (Agilent Technologies). Then, the hybridization was carried out using the Agilent 4 × 44 k Whole Human Genome microarrays (G4112F; Agilent Technologies). The hybridization experiment was completed in in the Agilent G2545A hybridization oven.

Hybridization signals were recorded by an Agilent G52565BA scanner and analyzed using Feature Extraction 9.3 (Agilent Technologies) and Spotfire 8.0 (Spotfire Inc., Cambridge, MA, USA) software. Spots that failed to pass the quality control procedures were flagged and excluded from further analysis. A possible dye-related bias in the microarray results was eliminated using an algorithm that involved application of normalization factors. The generated data were imported into spreadsheets (Excel; Microsoft, Redmond, WA, USA) for downstream data analysis and statistical evaluation. To identify differentially expressed genes, fold-change was calculated for each gene through dividing the fluorescent value of the KBD patient by that of the OA subject. Genes with fold-change ≥ 1.5 and *P* < 0.05 were regarded as differently expressed in KBD patients compared with OA patients. To further validate the microarray results, four upregulated genes (EPHA3, TRPC6, DOK5, and BIRC5) and four downregulated genes (BBC3, SMAD9, SLC25A37, and RASD1) were selected for quantitative reverse-transcription polymerase chain reaction (qRT-PCR) in our previous study [7]. The results of qRT-PCR experiment of the eight genes were entirely consistent with those of the microarray study, confirming the accuracy of the microarray experiment [7].

### Statistical analysis

In this study, InCroMAP software was applied for single-platform and cross-platform integrative pathway enrichment analysis [17]. The dataset sheet was formatted as required by InCroMAP . After importing the data into InCroMAP, data pairing was firstly performed to match the two datasets according to each probe’s identifier, and the genes which were commonly differentially expressed and methylated between KBD and OA were identified. Integrated gene set enrichment analysis was then conducted in InCroMAP by applying the threshold of *P*_*methy*_ < 0.05 and fold-change ≥ 1.5 [17].

## Results

### The main commonly differentially expressed and methylated genes

Six hundred and ninety-four genes were identified to be differently methylated between KBD and OA, including 285 hypermethylated CpG sites and 951 hypomethylated CpG sites, corresponding to 189 hypermethylated genes and 505 hypomethylated genes.

By performing the “pair-data” command in InCroMAP, 241 genes were identified to be commonly differentially expressed and methylated between KBD and OA. The family members of transforming growth factor (TGF) beta receptor, including TGFBR1 (NM_004612, fold-change = 2.077, *P*_*methy*_ = 0.0430), TGFBR2 (NM_001024847, fold-change = 1.543, *P*_*methy*_ = 0.037), and TGFBR3 (NM_001276, fold-change = 0.4515, *P*_*methy*_ = 6.04 × 10^−4^), were found to have different expression and methylation levels in KBD relative to OA. Additionally, CHST13 and ADAM12 were also identified to be commonly differentially expressed and methylated in KBD compared to OA (CHST13: NM_152889, fold-change = 0.5979, *P*_*methy*_ = 0.0430; ADAM12: NM_021641, fold-change = 1.9768, *P*_*methy*_ = 0.0178). Among these, TGFBR2 and ADAM12 showed increased mRNA expression levels and decreased DNA methylation levels in KBD cartilage compared with OA cartilage. TGFBR3 and CHST13 presented reduced mRNA expression and DNA methylation levels, while TGFBR1 showed increased mRNA expression and DNA methylation levels in KBD cartilage compared with OA cartilage. The main commonly differentially expressed and methylated genes can be seen in (Additional file 1: Table S1).

### Pathway enrichment analysis of DNA methylation data

Thirteen pathways were identified to be significantly enriched in the differentially methylated genes between KBD and OA cartilage (Additional file 2: Table S2), such as other types of O-glycan biosynthesis (path:hsa00514, *P* = 2.44 × 10^−3^), ABC transporters (path:hsa02010, *P* = 7.54 × 10^−3^), insulin secretion (path:hsa04911, *P* = 0.0132), mammalian target of rapamycin (mTOR) signaling pathway (path:hsa04150, *P* = 0.0144), and glycosaminoglycan (GAG) degradation (path:hsa00531, *P* = 0.0168).

### Integrative pathway enrichment analysis

Nineteen pathways were identified to be significantly altered regarding both expression and methylation levels (Table 1). The top five significant pathways included adherens junction (path:hsa04520, *P* = 5.34 × 10^−3^), insulin secretion (path:hsa04911, *P* = 7.32 × 10^−3^), other types of O-glycan biosynthesis (path:hsa00514, *P* = 0.0101), pathways in cancer (path:hsa05200, *P* = 0.0148), and pancreatic cancer (path:hsa05212, *P* = 0.0156).

**Table 1:** Integrative pathway enrichment analysis results of DNA methylation and mRNA expression profiles between KBD and OA cartilage

Pathway name	KEGG ID	*P* value	No. of significantly different genes*	No. of genes in KEGG pathway
Adherens junction	path:hsa04520	5.34 × 10^−3^	5	60
Insulin secretion	path:hsa04911	7.32 × 10^–3^	5	65
Other types of O-glycan biosynthesis	path:hsa00514	0.010	3	24
Pathways in cancer	path:hsa05200	0.015	10	247
Pancreatic cancer	path:hsa05212	0.016	4	52
Chronic myeloid leukemia	path:hsa05220	0.017	4	53
Valine, leucine and isoleucine biosynthesis	path:hsa00290	0.020	1	4
Type II diabetes mellitus	path:hsa04930	0.021	3	32
PI3K-Akt signaling pathway	path:hsa04151	0.024	9	233
Glycosaminoglycan biosynthesis—keratan sulfate	path:hsa00533	0.028	2	14
mTOR signaling pathway	path:hsa04150	0.030	3	37
Nicotinate and nicotinamide metabolism	path:hsa00760	0.031	2	15
Osteoclast differentiation	path:hsa04380	0.033	5	99
Glycosaminoglycan biosynthesis—chondroitin sulfate/dermatan sulfate	path:hsa00532	0.039	2	17
Transcriptional misregulation in cancer	path:hsa05202	0.041	6	143
Acute myeloid leukemia	path:hsa05221	0.047	3	45
Vascular smooth muscle contraction	path:hsa04270	0.048	4	77
Chagas disease (American trypanosomiasis)	path:hsa05142	0.048	4	77
Insulin signaling pathway	path:hsa04910	0.049	4	78

*KEGG* Kyoto Encyclopedia of Genes and Genomes, *mTOR* mammalian target of rapamycin, *PI3K* phosphatidylinositol 3’-kinase

* Number of genes differently expressed and methylated in Kashin-Beck disease (KBD) compared with osteoarthritis (OA) within the given pathway

**Fig. 1:**
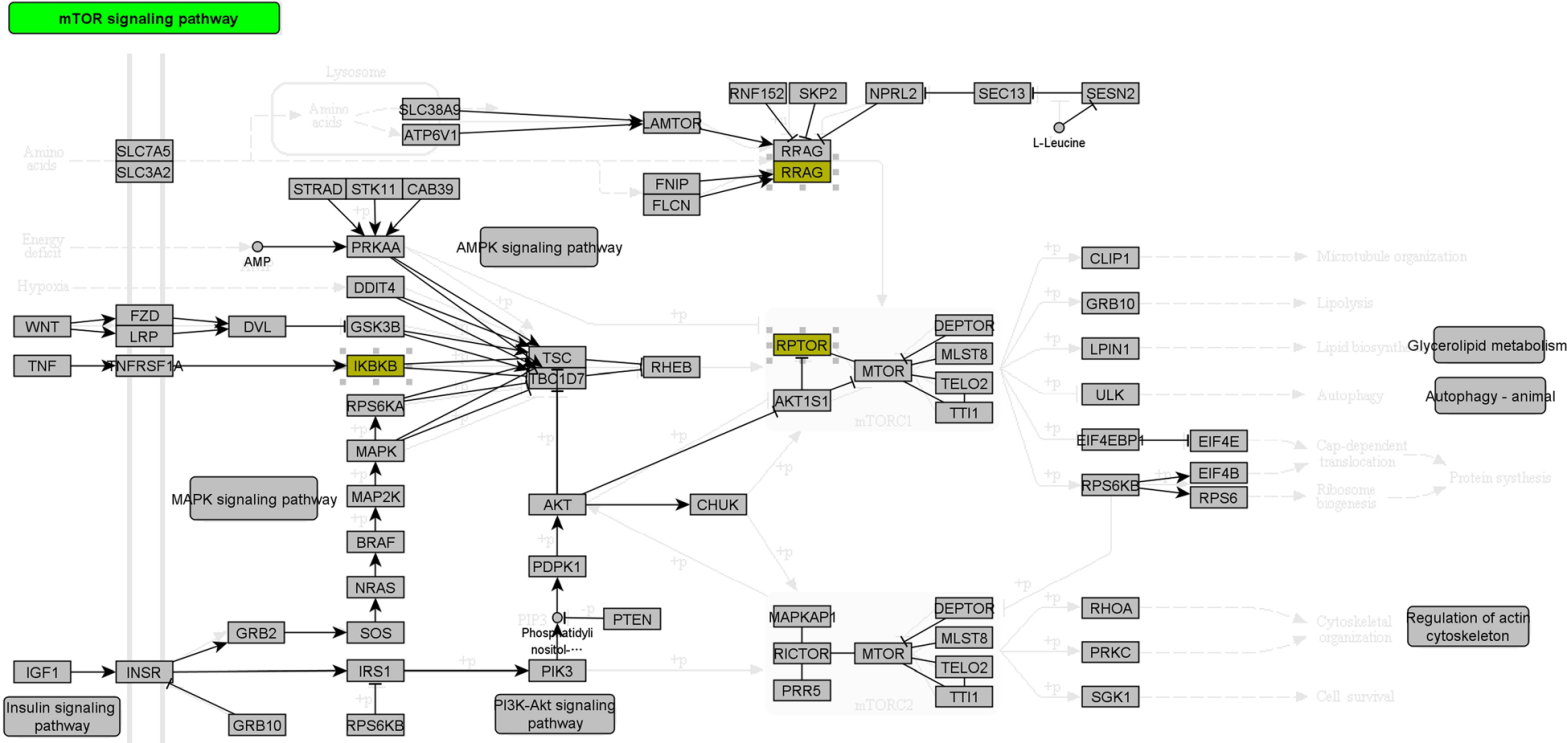
The KEGG pathway of mammalian target of rapamycin (mTOR) signaling pathway

The pathways related to glycosaminoglycan biosynthesis (path:hsa00532 and path:hsa00533) and the pathway related to osteoclast differentiation (path:hsa04380) were found to be altered significantly in KBD compared with OA (path:hsa00532, *P* = 0.0391; path:hsa00533, *P* = 0.0278; and path:hsa04380, *P* = 0.0325), and the mTOR signaling pathway (path:hsa04150) was also one of the significantly altered pathways (*P* = 0.0301; Fig. 1).

## Discussion

In this study, an integrative analysis of global DNA methylation and mRNA expression profiles was performed using InCroMAP; 241 genes were identified to be commonly differentially expressed and methylated between KBD and OA, and 19 pathways were found to be altered in KBD relative to OA.

The family of TGF-beta receptors was found to be commonly differentially expressed and methylated in KBD compared with OA, including TGFBR1, TGFBR2, TGFBR3 (Additional file 1: Table S1). TGF-beta can encode a secreted ligand of the TGF-beta superfamily of proteins and regulate cell differentiation and growth by binding to various TGF-beta receptors. TGFBR1 can form a heteromeric complex with TGFBR2 when bound to TGF-beta, transducing the TGF-beta signal from the cell surface to the cytoplasm. TGFBR3 is a membrane proteoglycan that often functions as a co-receptor with other TGF-beta receptor superfamily members. The TGF-beta superfamily consists of the TGF-beta subfamily and the bone morphogenetic protein subfamily. The balance between the two subfamilies is essential for the development of growth plate. In a previous study, TGF-beta was also found to have an enhanced expression level in the articular cartilage of KBD compared with that of normal cartilage [18]. Another study observed that after treatment with T-2 toxin, an important environmental risk for KBD, the chondrocytes had an elevated mRNA expression level of TGFB3 [19]. Therefore, based on others and our previous study results, we might infer that the disturbance of the TGF-beta superfamily induced by T-2 toxin led to the impaired development of the growth plate in KBD, but further biological studies were needed to confirm our finding. Additionally, TGF-beta has also played an important role in the development of OA by reducing collagen cleavage and chondrocyte differentiation [20]. However, the difference in the roles played by the TGF-beta superfamily in KBD and OA was still unclear.

We also found that ADAM12 showed increased mRNA expression levels and hypomethylated DNA levels in KBD cartilage compared to OA cartilage. ADAM12 belongs to the family of ADAMs (a disintegrin and metalloproteases). ADAM12 has been implicated in a variety of biological processes, involving cell-cell and cell-matrix interactions [21]. ADAM12-S transgenic mice were found to exhibit a pronounced increase in longitudinal bone growth and an increased number of proliferating chondrocytes [22]. In a previous genome-wide association study involving 2471 study subjects, ADAM12 polymorphism was identified to be significantly associated with joint destruction and growth retardation phenotypes of KBD and showed a decreased protein expression level in KBD cartilage compared to healthy control cartilage [23]. Given the impaired bone development in KBD children, ADAM12 might be involved in the skeletal growth retardation of KBD, but further biological studies were needed to reveal the roles of the identified genes in the development of KBD and OA.

Additionally, we observed that hypermethylated/hypomethylated genes did not always have lower/higher mRNA expression levels, such as TGFBR3 and CHST13. A similar phenomenon has been reported by previous studies [10, 24]. These results might be partly explained by the complicated transcriptional regulation of DNA methylation [25, 26].

The mTOR signaling pathway was one of the pathways which were found to be altered on the gene expression and DNA methylation scales in KBD relative to OA. mTOR is a highly conversed serine/threonine protein kinase that can integrate stimuli, such as nutrients and growth factors, to regulate several biological processes, including metabolism, autophagy, protein synthesis, and ribosome biogenesis. mTOR can be inhibited by rapamycin and induce the autophagy process [27]. Recently, the important role of mTOR signaling in the progression of OA was shown in several studies [28, 29]. Intra-articular injection of rapamycin could attenuate the articular cartilage degradation caused by OA through reducing the expression of mTOR [29]. The cartilage-specific ablation of mTOR in a mouse model could result in increased autophagy signaling and a significant protection from medial meniscus-induced OA [28]. Additionally, mTOR signaling was also thought to be related to skeletal growth in mammals. Genetic deletion of mTOR1 or mTOR2, the two distinct complexes of mTOR, could diminish embryonic skeletal growth due to severe delays in chondrocyte hypertrophy and impaired bone formation [30, 31].

However, little was previously known about mTOR signaling in KBD chondrocytes. For the first time, we found the mTOR signaling pathway to be altered in KBD chondrocytes compared with OA in this study. A previous study identified that defective autophagy could be seen in KBD chondrocytes with reduced Beclin1 and LC3, and several autophagy-related genes were found to be differentially expressed in KBD chondrocytes compared with the normal control [32]. Due to the significant effects of mTOR signaling on the autophagy process and skeletal growth, it was considered worthy to further study mTOR in KBD chondrocytes.

The pathways related to the glycosaminoglycan (GAG) biosynthesis, including chondroitin sulfate (CS)/dermatan sulfate (DS) and keratan sulfate (KS), were also found to be altered on the gene expression and DNA methylation scales in KBD chondrocytes relative to OA. GAGs, a heterogeneous family of unbranched polysaccharides, exist either as a free state or attached to proteins to form proteoglycans (PGs) and can be found both on the cell surface and in the extracellular matrix (ECM) [33]. PGs and GAGs are the ECM components of articular cartilage, whose functions are critical for the articular cartilage [34]. There are three classes of GAGs in cartilage, including hyaluronan (HA), CS, and KS. Since DS is a modified form of CS, DS was included into the CS class here. CS can account for 80% of GAGs and KS can account for 5–20% of GAGs in articular cartilage [35]. They together provide cartilage resistance to the physical stress and load [35].

Previous studies have shown that CS and KS dramatically decrease in the articular cartilage of KBD children compared with normal controls [36]. Urine hydroxyproline, a marker of catabolism levels of PGs in vivo, was also higher in children from the KBD endemic areas [37]. Moreover, a series of enzymes involved in CS metabolism were identified to be abnormally expressed in KBD cartilage—for example, the anabolic enzymes including PAPSS2, PAPST1, and CHST15 were significantly lower in KBD samples than those found in the controls; in contrast, the catabolic enzymes including ARSB and GALNS were significantly higher than in the control samples [38]. This evidence suggests a global disruption of glycosaminoglycan biosynthesis in KBD cartilage. In this study, CHST13, another kind of sulfotransferase responsible for the anabolism of CS, was also found to be lower in KBD chondrocytes relative to OA. Additionally, an abnormal DNA methylation level of other genes related to glycosaminoglycan biosynthesis, such as UST, was also identified in this study. Because of the lack of DNA methylation studies in KBD chondrocytes, how DNA methylation regulates the expression of GAG-related genes remains unknown, and further efforts should be made to study this field.

Interestingly, the pathway for osteoclast differentiation was also found to be altered in the cartilage in this study. The pathway for osteoclast differentiation can regulate the genesis of osteoclasts, a kind of multinucleate cell originating from the hematopoietic monocyte-macrophage lineage and responsible for bone resorption. The dynamic balance of bone-forming osteoblasts and bone-resorbing osteoclasts is essential for the homeostasis of the bony skeleton. Impaired osteoclasts can result in osteoporosis (OP) [39]. KBD, but not OA, is characteristic of OP [40]. In addition, KBD and OP phenotypes were found to have pleiotropic effects and share common risk genes in a previous study [40]. However, this study was conducted in cartilage chondrocytes but not in osteoclasts, and it lacked further evidence to conclude what role these osteoclast differentiation-related genes played in chondrocytes and what the situation was regarding KBD osteoclasts.

The phosphatidylinositol 3’-kinase (PI3K)-Akt signaling pathway was another altered pathway identified in this study. The PI3K-Akt signaling pathway can regulate fundamental cellular functions such as cell growth, survival, and movement. If this pathway is inhibited in chondrocytes, the cells show a reduced level of survival and proteoglycan synthesis [41]. This pathway was additionally thought to be a potential target for the treatment of OA [42–44]. In a previous study conducted in KBD chondrocytes compared with normal controls, the PI3K-Akt signaling pathway was identified to be significantly differentially expressed, and it was concluded that an environmental risk factor for KBD, selenium deficiency, might induce chondrocyte apoptosis and cell death through the PI3K-Akt signaling pathway [45]. Moreover, in this study, the PI3K-Akt signaling pathway showed different levels of gene expression and DNA methylation in KBD cartilage relative to OA. Based on previous studies and our study results, we suggest that the PI3K-Akt pathway exerts its effect in different ways in KBD compared with OA. However, the interaction of genes within the PI3K-Akt pathway is complicated and needs further study.

There were some limitations to this study. Firstly, articular cartilage specimens were collected from severe KBD and OA patients undergoing total joint arthroplasty. Currently, there are limited numbers of severe KBD patients who need surgical treatment [46]. Due to articular cartilage degradation, the amount of DNA and mRNA samples extracted from collected cartilage specimens is generally not enough for both genome-wide DNA methylation and mRNA expression profiling experiments. Therefore, a relative small sample of five KBD and five OA patients was used for DNA methylation profiling and another independent sample of four KBD patients and four OA patients was used for mRNA expression profiling. A similar sampling design has been used by previous studies [47, 48]. Secondly, because it was impossible to collect articular cartilage specimens from early-stage patients all the study subjects were at the end-stage of KBD or OA. Therefore, our study results trend to reflect the alterations in end-stage disease. Third, utilizing the pathway enrichment analysis approach of InCroMAP software, we identified multiple pathways that were significantly enriched in the differentially expressed and differentially methylated gene sets between KBD and OA. However, according to the pathway enrichment analysis results of InCroMAP, we could not determine whether the identified pathways were activated or inhibited. Further biological studies are warranted to reveal the mechanisms of the identified pathways implicated in the development of KBD and OA.

In summary, this was the first integrative study in KBD combining gene expression profiling and DNA methylation profiling datasets. By performing integrative enrichment analysis with two datasets, 19 pathways were identified to be altered in KBD cartilage relative to OA. For the first time, the autophagy and ECM metabolism-related pathways (mTOR signaling pathway, glycosaminoglycan biosynthesis) were especially found to be differentially methylated and expressed in KBD relative to OA.

## Conclusion

This study suggests a different molecular feature in the pathology of KBD and OA and provides new clues for the study of KBD and OA.

## Additional files

Additional file 1: **Table S1.** The common significant genes detected by both DNA methylation and mRNA expression profiling studies. (DOCX 26 kb)

Additional file 2: **Table S2.** Pathway enrichment analysis results of genome-wide DNA methylation profiling. (DOCX 16 kb)

## Abbreviations

CS: Chondroitin sulfate
DS: Dermatan sulfate
ECM: Extracellular matrix
FDR: False discovery rate
GAG: Glycosaminoglycan
HA: Hyaluronan
KBD: Kashin-Beck disease
KS: Keratan sulfate
mTOR: Mammalian target of rapamycin
OA: Osteoarthritis
OP: Osteoporosis
PG: Proteoglycan
PI3K: Phosphatidylinositol 3′-kinase
qRT-PCR: Quantitative reverse-transcription polymerase chain reaction
TGF: Transforming growth factor
