# Supplementary material for: Integrating genome-wide DNA methylation and mRNA expression profiles identified different molecular features between Kashin-Beck disease and primary osteoarthritis
Source: Arthritis Res Ther. 2018 Mar 7;20:41. doi: 10.1186/s13075-018-1531-1 (PMC5842623; doi:10.1186/s13075-018-1531-1)
Supplement: Supplementary file 1 — Table S1. The common significant genes detected by both DNA methylation and mRNA expression profiling studies. (DOCX 26 kb) [file 13075_2018_1531_MOESM1_ESM.docx]

**Table S1.** The common significant genes detected by both DNA methylation and mRNA expression profiling studies

| **Gene Symbol** | **Ref Seq** | **Chromosome** | ***P* Value of DNA Methylation** | **Fold Change* of mRNA Expression** |
| --- | --- | --- | --- | --- |
| ABCA6 | NM_080284 | chr17 | 5.56E-03 | 2.4524 |
| ACTL8 | NM_030812 | chr1 | 3.31E-03 | 1.8442 |
| ADAM12 | NM_021641, NM_003474 | chr10 | 0.0178 | 1.9768 |
| ADAM32 | NM_145004 | chr8 | 0.0247 | 2.1515 |
| ADAMTSL1 | NM_052866, NM_001040272 | chr9 | 6.17E-03 | 2.1062 |
| ADAMTSL2 | NM_014694 | chr9 | 0.018 | 0.666 |
| AGPAT4 | NM_020133 | chr6 | 0.0308 | 0.55 |
| AK5 | NM_174858 | chr1 | 0.0346 | 1.7443 |
| ANGPT1 | NM_001146 | chr8 | 0.0198 | 6.6279 |
| ANK1 | NM_020478, NM_000037 | chr8 | 0.0203 | 5.4578 |
| ANK3 | NM_001149, NM_020987 | chr10 | 1.61E-04 | 5.7034 |
| ANKRD11 | NM_013275 | chr16 | 9.98E-03 | 1.6761 |
| ANO4 | NM_178826 | chr12 | 0.02 | 3.9748 |
| ANO8 | NM_020959 | chr19 | 0.011 | 1.902 |
| ANXA11 | NM_145869 | chr10 | 3.02E-03 | 2.2361 |
| ANXA5 | NM_001154 | chr4 | 0.0245 | 1.8729 |
| APBB2 | NM_173075 | chr4 | 0.042 | 2.2401 |
| AQP1 | NM_198098 | chr7 | 0.0332 | 7.4698 |
| ARG2 | NM_001172 | chr14 | 8.16E-03 | 2.8811 |
| ARID5B | NM_032199 | chr10 | 0.0188 | 2.1726 |
| ARL4C | NM_005737 | chr2 | 4.54E-03 | 0.3626 |
| ARNTL2 | NM_020183 | chr12 | 0.0484 | 2.0603 |
| ATP6V0A4 | NM_020632 | chr7 | 0.011 | 1.97 |
| ATP6V1A | NM_001690 | chr3 | 7.66E-03 | 0.6285 |
| AUTS2 | NM_015570 | chr7 | 0.0299 | 2.6273 |
| BBS2 | NM_031885 | chr16 | 0.0174 | 1.6302 |
| BBS9 | NM_198428 | chr7 | 3.19E-03 | 3.9045 |
| BCAT1 | NM_005504 | chr12 | 0.0177 | 1.6608 |
| BCL9 | NM_004326 | chr1 | 9.04E-03 | 0.6256 |
| BICD1 | NM_001003398 | chr12 | 0.0105 | 3.4696 |
| BLCAP | NM_006698 | chr20 | 0.0187 | 1.513 |
| BOC | NM_033254 | chr3 | 1.47E-03 | 0.5818 |
| BPGM | NM_199186 | chr7 | 3.08E-03 | 2.1176 |
| BSN | NM_003458 | chr3 | 8.73E-03 | 1.5353 |
| BST1 | NM_004334 | chr4 | 0.0203 | 3.7597 |
| C10ORF11 | NM_032024 | chr10 | 0.0128 | 2.0703 |
| C18ORF45 | NM_032933 | chr18 | 6.26E-04 | 1.7743 |
| C21ORF7 | NM_020152 | chr21 | 6.46E-03 | 2.0914 |
| C6ORF162 | NM_020425 | chr6 | 0.0108 | 2.7318 |
| C8ORF42 | NM_175075 | chr8 | 7.56E-03 | 2.274 |
| C8ORF84 | NM_153225 | chr8 | 0.0104 | 5.9607 |
| CACNA1C | NM_000719 | chr12 | 0.0138 | 2.3052 |
| CAPN2 | NM_001748 | chr1 | 0.022 | 2.1312 |
| CCDC66 | NM_001012506 | chr3 | 3.85E-03 | 1.5886 |
| CCDC68 | NM_025214 | chr18 | 0.0151 | 1.6119 |
| CCL26 | NM_006072 | chr7 | 0.023 | 1.5618 |
| CD28 | NM_006139 | chr2 | 1.88E-04 | 2.8363 |
| CDCP1 | NM_022842, NM_178181 | chr3 | 0.0251 | 3.6906 |
| CDK14 | NM_012395 | chr7 | 0.0104 | 2.8623 |
| CGNL1 | NM_032866 | chr15 | 2.33E-03 | 0.5563 |
| CHCHD6 | NM_032343 | chr3 | 0.0395 | 0.6639 |
| CHI3L1 | NM_001276 | chr1 | 0.0444 | 0.4599 |
| CHST13 | NM_152889 | chr3 | 0.043 | 0.5979 |
| CLTCL1 | NM_007098 | chr22 | 4.04E-03 | 1.5484 |
| CNN3 | NM_001839 | chr1 | 0.0412 | 1.7639 |
| CNST | NM_152609 | chr1 | 0.0158 | 1.7326 |
| CORO2B | NM_006091 | chr15 | 0.0202 | 2.0405 |
| CREB5 | NM_182898 | chr7 | 0.0113 | 1.733 |
| DAPK2 | NM_014326 | chr15 | 0.0206 | 2.0715 |
| DAXX | NM_001350 | chr6 | 0.0103 | 0.5853 |
| DCLK1 | NM_004734 | chr13 | 0.0251 | 16.8512 |
| DCN | NM_001920 | chr12 | 3.76E-03 | 3.1103 |
| DHX58 | NM_024119 | chr17 | 0.0341 | 0.6653 |
| DLGAP4 | NM_014902 | chr20 | 2.97E-03 | 1.9129 |
| DOCK1 | NM_001380 | chr10 | 0.0157 | 2.8194 |
| DOCK9 | NM_015296 | chr13 | 0.0351 | 1.7261 |
| EBF3 | NM_001005463 | chr10 | 4.01E-03 | 3.6162 |
| EFCAB6 | NM_022785 | chr22 | 0.0219 | 2.0358 |
| EPN2 | NM_014964 | chr17 | 0.0191 | 0.6066 |
| EPS8 | NM_004447 | chr12 | 0.0497 | 1.9886 |
| ESPNL | NM_194312 | chr2 | 0.0231 | 5.2757 |
| FAM196A | NM_001039762 | chr10 | 0.0157 | 1.9201 |
| FAM20A | NM_017565 | chr17 | 2.07E-03 | 3.6436 |
| FAT1 | NM_005245 | chr4 | 0.0356 | 2.8093 |
| FBN2 | NM_001999 | chr5 | 2.45E-03 | 7.2515 |
| FMN2 | NM_020066 | chr1 | 1.17E-03 | 15.4119 |
| FMNL3 | NM_175736 | chr12 | 2.58E-03 | 2.4791 |
| FPR1 | NM_002029 | chr19 | 0.0105 | 1.7576 |
| GALNTL4 | NM_198516 | chr11 | 0.0384 | 2.1947 |
| GDPD3 | NM_024307 | chr16 | 0.0372 | 0.5335 |
| GRAMD3 | NM_023927 | chr5 | 4.03E-03 | 1.9385 |
| GRASP | NM_181711 | chr12 | 2.18E-03 | 1.6828 |
| GULP1 | NM_016315 | chr2 | 0.0209 | 1.8025 |
| HDAC4 | NM_006037 | chr2 | 1.45E-03 | 1.5516 |
| HIBCH | NM_014362 | chr2 | 1.57E-03 | 2.0295 |
| HKDC1 | NM_025130 | chr10 | 8.42E-03 | 1.9875 |
| HLX | NM_021958 | chr1 | 0.0168 | 0.5585 |
| HPS3 | NM_032383 | chr3 | 4.52E-03 | 1.6349 |
| IFI16 | NM_005531 | chr1 | 0.0193 | 2.3869 |
| IGDCC4 | NM_020962 | chr15 | 0.0139 | 1.5353 |
| IGF2BP3 | NM_006547 | chr7 | 0.0162 | 6.125 |
| INSR | NM_000208 | chr19 | 0.0303 | 0.6641 |
| ITGBL1 | NM_004791 | chr13 | 0.0261 | 4.9413 |
| JAK1 | NM_002227 | chr1 | 3.97E-03 | 1.5282 |
| JARID2 | NM_004973 | chr6 | 9.34E-03 | 2.7863 |
| KALRN | NM_003947, NM_001024660 | chr3 | 6.00E-04 | 5.3813 |
| KCNB1 | NM_004975 | chr20 | 5.70E-03 | 9.3802 |
| KCNS3 | NM_002252 | chr2 | 2.31E-03 | 3.0402 |
| KCTD16 | NM_020768 | chr5 | 5.72E-03 | 4.9636 |
| KDM2A | NM_012308 | chr11 | 7.42E-03 | 0.6181 |
| KIAA1704 | NM_018559 | chr13 | 0.0241 | 2.8705 |
| KIF7 | NM_198525 | chr15 | 0.0261 | 2.1508 |
| KLF6 | NM_001300 | chr10 | 8.20E-03 | 3.3944 |
| KRT80 | NM_182507 | chr12 | 1.08E-03 | 2.7191 |
| LAMC1 | NM_002293 | chr1 | 4.41E-03 | 1.9469 |
| LARS2 | NM_015340 | chr3 | 1.97E-03 | 1.5075 |
| LBH | NM_030915 | chr2 | 0.0474 | 1.6214 |
| LCLAT1 | NM_182551 | chr2 | 8.49E-03 | 2.4824 |
| LDB2 | NM_001290 | chr4 | 7.47E-03 | 4.3058 |
| LFNG | NM_001040167, NM_001040168 | chr7 | 7.92E-03 | 3.5202 |
| LHPP | NM_022126 | chr10 | 0.0118 | 6.1288 |
| LIMS1 | NM_004987 | chr2 | 3.36E-03 | 1.7687 |
| LIPC | NM_000236 | chr15 | 0.0116 | 1.6459 |
| LMO2 | NM_005574 | chr11 | 0.0176 | 1.6508 |
| LPAR1 | NM_057159 | chr9 | 0.0279 | 1.7799 |
| LRIG1 | NM_015541 | chr3 | 6.79E-03 | 0.4756 |
| LRRC8C | NM_032270 | chr1 | 0.0494 | 2.0366 |
| LSAMP | NM_002338 | chr3 | 0.0201 | 2.0643 |
| LTBP1 | NM_206943 | chr2 | 0.0228 | 2.2734 |
| LYPD6B | NM_177964 | chr2 | 0.0278 | 4.2447 |
| MAMDC2 | NM_153267 | chr9 | 8.72E-03 | 4.291 |
| MAPRE1 | NM_012325 | chr20 | 3.32E-04 | 1.7417 |
| MCC | NM_002387 | chr5 | 0.0117 | 1.586 |
| MED1 | NM_004774 | chr17 | 0.05 | 1.8325 |
| METAP1D | NM_199227 | chr2 | 1.20E-04 | 3.7718 |
| MFAP2 | NM_017459 | chr1 | 0.0323 | 2.404 |
| MGAT5B | NM_144677 | chr17 | 0.0173 | 1.7591 |
| MSRA | NM_012331 | chr8 | 0.0116 | 3.3918 |
| MTMR2 | NM_201278 | chr11 | 0.0494 | 2.4226 |
| MYLK | NM_053025 | chr3 | 3.24E-03 | 5.7689 |
| NAV3 | NM_014903 | chr12 | 0.0424 | 45.5072 |
| NFIB | NM_005596 | chr9 | 3.96E-03 | 2.2924 |
| NGEF | NM_019850 | chr2 | 6.92E-03 | 2.2257 |
| NLRP5 | NM_153447 | chr19 | 0.0327 | 1.6588 |
| NMNAT2 | NM_015039 | chr1 | 0.0142 | 1.6902 |
| NPHP4 | NM_015102 | chr1 | 0.0418 | 1.5962 |
| NPTN | NM_012428 | chr15 | 0.0165 | 1.6082 |
| NRP1 | NM_003873 | chr10 | 0.014 | 2.4366 |
| NTRK3 | NM_001012338, NM_002530 | chr15 | 3.63E-03 | 0.6523 |
| NUFIP1 | NM_012345 | chr13 | 0.0241 | 1.739 |
| OLFM1 | NM_006334, NM_014279 | chr9 | 0.0257 | 3.6975 |
| PALLD | NM_016081 | chr4 | 0.031 | 2.8483 |
| PARVA | NM_018222 | chr11 | 0.0361 | 1.7696 |
| PCBP3 | NM_020528 | chr21 | 0.0168 | 1.7333 |
| PCSK2 | NM_002594 | chr20 | 0.0229 | 2.6244 |
| PCSK6 | NM_002570, NM_138322, NM_138325 | chr15 | 0.0396 | 1.5299 |
| PDE4B | NM_001037341, NM_002600, NM_001037339 | chr1 | 4.40E-03 | 0.5176 |
| PDE4D | NM_006203 | chr5 | 0.0156 | 1.6776 |
| PHF17 | NM_024900 | chr4 | 3.33E-03 | 0.435 |
| PLEKHA5 | NM_019012 | chr12 | 0.0118 | 2.6436 |
| PLXDC1 | NM_020405 | chr17 | 5.47E-03 | 1.8134 |
| PPAPDC1B | NM_032483 | chr8 | 7.19E-04 | 2.341 |
| PPIF | NM_005729 | chr10 | 0.0404 | 0.5761 |
| PRRT1 | NM_030651 | chr6 | 0.0204 | 0.6484 |
| PRTFDC1 | NM_020200 | chr10 | 0.0165 | 3.1432 |
| PTN | NM_002825 | chr7 | 9.05E-04 | 18.0741 |
| PXDN | NM_012293 | chr2 | 0.017 | 3.257 |
| PXK | NM_017771 | chr3 | 9.90E-03 | 1.57 |
| RAMP1 | NM_005855 | chr2 | 0.0215 | 2.2189 |
| RARB | NM_000965 | chr3 | 0.0146 | 2.1746 |
| RBMS1 | NM_002897, NM_016836 | chr2 | 8.38E-04 | 1.8876 |
| RBMS3 | NM_001003793, NM_014483 | chr3 | 7.16E-03 | 4.6595 |
| RFTN1 | NM_015150 | chr3 | 8.04E-03 | 3.2603 |
| RFX2 | NM_000635 | chr19 | 0.0122 | 2.3115 |
| ROR1 | NM_005012 | chr1 | 2.08E-03 | 2.8613 |
| ROR2 | NM_004560 | chr9 | 9.87E-04 | 1.8005 |
| RORA | NM_134260 | chr15 | 0.0221 | 2.6232 |
| RPTOR | NM_020761 | chr17 | 0.0242 | 2.1702 |
| RRAGD | NM_021244 | chr6 | 1.24E-03 | 1.6114 |
| RSPH3 | NM_031924 | chr6 | 6.05E-03 | 1.9222 |
| RUNX1 | NM_001001890 | chr21 | 5.26E-03 | 2.9877 |
| RUNX2 | NM_001015051 | chr6 | 7.07E-03 | 1.754 |
| RUVBL1 | NM_003707 | chr3 | 8.70E-03 | 2.6116 |
| S100B | NM_006272 | chr21 | 2.45E-03 | 1.6621 |
| SDCCAG8 | NM_006642 | chr1 | 4.80E-03 | 1.9933 |
| SDHC | NM_003001 | chr1 | 0.0143 | 1.5719 |
| SEL1L3 | NM_015187 | chr4 | 0.0143 | 9.5175 |
| SEMA4F | NM_004263 | chr2 | 0.0203 | 1.7684 |
| SH3PXD2A | NM_014631 | chr10 | 0.0358 | 1.5294 |
| SHANK2 | NM_133266, NM_012309 | chr11 | 5.79E-03 | 9.6158 |
| SHFM1 | NM_006304 | chr7 | 6.22E-03 | 1.6189 |
| SIPA1L1 | NM_015556 | chr14 | 7.79E-03 | 1.7114 |
| SLC26A4 | NM_000441 | chr7 | 0.0326 | 3.5369 |
| SLC2A1 | NM_006516 | chr1 | 2.59E-03 | 2.3534 |
| SLC36A2 | NM_181776 | chr5 | 4.70E-03 | 1.5255 |
| SLC6A6 | NM_003043 | chr3 | 0.0229 | 0.586 |
| SLC9A9 | NM_173653 | chr3 | 3.26E-03 | 1.7983 |
| SLPI | NM_003064 | chr20 | 3.08E-03 | 0.3212 |
| SMAD3 | NM_005902 | chr15 | 2.67E-03 | 1.7048 |
| SNX6 | NM_021249 | chr14 | 0.0301 | 1.8228 |
| SORBS1 | NM_015385, NM_006434 | chr10 | 9.01E-03 | 2.0879 |
| SORCS2 | NM_020777 | chr4 | 0.0323 | 5.4936 |
| SPARCL1 | NM_004684 | chr4 | 3.87E-04 | 8.2776 |
| SSPN | NM_005086 | chr12 | 0.0173 | 1.8462 |
| SSR3 | NM_007107 | chr3 | 0.0406 | 2.0898 |
| ST3GAL1 | NM_003033 | chr8 | 0.0104 | 0.4271 |
| ST5 | NM_005418 | chr11 | 0.0197 | 0.5929 |
| STK39 | NM_013233 | chr2 | 3.40E-03 | 4.7785 |
| STMN2 | NM_007029 | chr8 | 2.23E-04 | 14.9593 |
| SULF1 | NM_015170 | chr8 | 5.88E-03 | 16.627 |
| SUPT3H | NM_003599 | chr6 | 8.35E-03 | 2.2798 |
| SYNC | NM_030786 | chr1 | 3.56E-03 | 7.2035 |
| SYNM | NM_145728 | chr15 | 8.16E-03 | 2.0925 |
| TBCD | NM_005993 | chr17 | 0.0253 | 2.1432 |
| TCF7L1 | NM_031283 | chr2 | 0.0105 | 1.6485 |
| TGFBR1 | NM_004612 | chr9 | 0.0196 | 2.0773 |
| TGFBR2 | NM_001024847, NM_003242 | chr3 | 0.037 | 1.5432 |
| TGFBR3 | NM_003243 | chr1 | 6.04E-04 | 0.4515 |
| THBS2 | NM_003247 | chr6 | 2.51E-04 | 2.5914 |
| THSD4 | NM_024817 | chr15 | 8.22E-03 | 3.061 |
| TIMP2 | NM_003255 | chr17 | 0.0326 | 0.6423 |
| TK1 | NM_003258 | chr17 | 0.0362 | 3.0108 |
| TLE4 | NM_007005 | chr9 | 0.0393 | 2.3474 |
| TMEM119 | NM_181724 | chr12 | 5.74E-03 | 1.699 |
| TMEM154 | NM_152680 | chr4 | 0.0309 | 2.1001 |
| TMTC2 | NM_152588 | chr12 | 1.34E-03 | 2.7034 |
| TMX1 | NM_030755 | chr14 | 0.0263 | 2.4639 |
| TPCN2 | NM_139075 | chr11 | 7.52E-03 | 2.5362 |
| TREM1 | NM_018643 | chr6 | 0.0105 | 2.2427 |
| TRIP13 | NM_004237 | chr5 | 0.046 | 3.1764 |
| TRPM4 | NM_017636 | chr19 | 0.0161 | 0.6036 |
| TRPV2 | NM_016113 | chr17 | 7.61E-03 | 3.4627 |
| TSPAN15 | NM_012339 | chr10 | 0.0146 | 4.1671 |
| TULP4 | NM_020245 | chr6 | 0.0346 | 1.9866 |
| UAP1L1 | NM_207309 | chr9 | 7.22E-03 | 0.6335 |
| UCN2 | NM_033199 | chr3 | 4.31E-03 | 0.5652 |
| UST | NM_005715 | chr6 | 0.0239 | 2.3252 |
| VAMP5 | NM_006634 | chr2 | 0.0123 | 1.646 |
| VGLL4 | NM_014667 | chr3 | 3.19E-03 | 0.5988 |
| VIM | NM_003380 | chr10 | 0.0191 | 3.4359 |
| VWA5A | NM_014622, NM_198315 | chr11 | 1.74E-03 | 2.3089 |
| WDR1 | NM_017491 | chr4 | 2.33E-03 | 2.229 |
| WWOX | NM_016373, NM_130791 | chr16 | 5.39E-03 | 1.9483 |
| ZC3H3 | NM_015117 | chr8 | 0.0153 | 0.6073 |
| ZDHHC14 | NM_153746 | chr6 | 0.0482 | 1.6674 |
| ZDHHC3 | NM_016598 | chr3 | 0.0185 | 2.0511 |
| ZIC1 | NM_003412 | chr3 | 4.56E-03 | 7.8655 |
| ZIC4 | NM_032153 | chr3 | 1.40E-03 | 2.0141 |
| ZNF335 | NM_022095 | chr20 | 0.0112 | 0.5148 |
| ZNF365 | NM_014951 | chr10 | 0.047 | 2.0081 |
| ZNF423 | NM_015069 | chr16 | 1.04E-03 | 2.0978 |

***Note:*** * The fold change denotes the ratios of mRNA expression levels between KBD and OA.
