# Supplementary material for: Integrating genome-wide DNA methylation and mRNA expression profiles identified different molecular features between Kashin-Beck disease and primary osteoarthritis
Source: Arthritis Res Ther. 2018 Mar 7;20:41. doi: 10.1186/s13075-018-1531-1 (PMC5842623; doi:10.1186/s13075-018-1531-1)
Supplement: Supplementary file 2 — Table S2. Pathway enrichment analysis results of genome-wide DNA methylation profiling. (DOCX 16 kb) [file 13075_2018_1531_MOESM2_ESM.docx]

**Table S2.** Pathway enrichment analysis results of genome-wide DNA methylation profile

| **Pathway name** | **KEGG ID** | ***P* value** | **No. of differently methylated genes*** | **No. of genes in KEGG pathway** |
| --- | --- | --- | --- | --- |
| Other types of O-glycan biosynthesis | path:hsa00514 | 2.44×10^-3^ | 5 | 24 |
| ABC transporters | path:hsa02010 | 7.54×10^-3^ | 6 | 44 |
| Insulin secretion | path:hsa04911 | 0.0132 | 7 | 65 |
| mTOR signaling pathway | path:hsa04150 | 0.0144 | 5 | 37 |
| Glycosaminoglycan degradation | path:hsa00531 | 0.0168 | 3 | 14 |
| Transcriptional misregulation in cancer | path:hsa05202 | 0.0213 | 11 | 143 |
| Shigellosis | path:hsa05131 | 0.0266 | 5 | 44 |
| Adherens junction | path:hsa04520 | 0.027 | 6 | 60 |
| Vascular smooth muscle contraction | path:hsa04270 | 0.0274 | 7 | 77 |
| Glycosaminoglycan biosynthesis - chondroitin sulfate / dermatan sulfate | path:hsa00532 | 0.0276 | 3 | 17 |
| Thyroid hormone synthesis | path:hsa04918 | 0.033 | 5 | 47 |
| Type II diabetes mellitus | path:hsa04930 | 0.0336 | 4 | 32 |
| Bacterial invasion of epithelial cells | path:hsa05100 | 0.0401 | 5 | 50 |

*Note*: * the number of genes differently methylated between KBD and OA within a given KEGG pathway.
